# Regulation of antibiotic sales in Mexico: an analysis of printed media coverage and stakeholder participation

**DOI:** 10.1186/1471-2458-12-1051

**Published:** 2012-12-06

**Authors:** Anahí Dreser, Edna Vázquez-Vélez, Sandra Treviño, Veronika J Wirtz

**Affiliations:** 1 Center for Health Systems Research, National Institute of Public Health, Av. Universidad 655, Cuernavaca, Mor, C.P. 62100, Mexico

**Keywords:** Antibiotics, Mexico, Over-the-counter sales, Antibiotic resistance, Media analysis, Pharmaceutical policy

## Abstract

**Background:**

Restricting antibiotics sales to those with medical prescriptions only is a central strategy for promoting appropriate use and containing antibiotic resistance; however, many low and middle income countries have not enforced policies that prevent widespread self-medication with antibiotics. In 2010, the Mexican government announced the enforcement of antibiotic sales regulations, a policy that gained media prominence. This study analyzes media coverage of issues, stakeholder representation, and positions taken during policy agenda setting, drafting, and implementation to shed light on policy making to promote appropriate antibiotic utilization.

**Methods:**

We carried out a quantitative content analysis of 322 newspaper articles published between January 2009 and December 2010 in 18 national and regional newspapers. Additionally, we conducted a qualitative content analysis to understand the positions adopted and strategies developed by nine key stakeholders. Framing theory guided the analysis.

**Results:**

The Ministry of Health dominated media coverage, justifying the enforcement policy by focusing on risks of self-medication, and to a lesser degree dangers of increasing antibiotic resistance. Pharmacy associations appeared to be the leading opponents, arguing that the policy created logistical difficulties and corruption, and had negative economic impact for pharmacies and their clients. The associations developed strategies against the regulation such as attempting to delay implementation and installing physicians’ consultation offices within pharmacies. While medical associations and academic institutions called for a comprehensive strategy to combat antibiotic resistance, improve prescription quality, and create public awareness, these issues had little impact on media coverage. Consumer groups and legislators received very little media coverage.

**Conclusions:**

The narrowly-focused and polarized media coverage ─centred on problems of self- medication and economic impact ─ was a missed opportunity to publicly discuss and to develop a comprehensive national strategy on antibiotic use in Mexico. It highlights the need for discussing and developing interventions within the framework of a pharmaceutical policy.

## Background

Aiming to address the global public health threat caused by antimicrobial resistance, the World Health Assembly in 1998 urged member countries to promote the appropriate use of antimicrobials by improving prescribing, dispensing, surveillance and legislation [1]. In the same year, the Pan American Conference on Antimicrobial Resistance in the Americas recommended that countries improve antibiotic use, including restriction on antibiotic sales to prescription-only and education on the proper use of antibiotics for health professionals and community members [2]. In 2001, the World Health Organization (WHO) launched its Global Strategy for Containment of Antimicrobial Resistance. Recognizing that isolated interventions have little impact on improving antibiotic use, the Global Strategy proposed a range of interventions, organized under the umbrella of national health and medicine policies [3]. These recommendations were updated in 2011 [4].

Only a few, largely high-income, countries have developed national strategies to promote appropriate antibiotic use; many countries have had difficulty translating international recommendations into national action plans. In part, difficulties have resulted from multiple stakeholder coordination, rigid legal frameworks, and potential conflicts between guaranteeing access to medicines and assuring rational use [5,6].

### Antibiotic policies in Mexico

Mexico is a middle-income country characterized by a fragmented health care system; expenditure in medicines occurs largely as out-of-pocket [7]. Until recently, antibiotics were among medicines most commonly sold in private pharmacies (approximately 40% without prescription) [8], and their use for many years exceeded that of other Latin American countries [9]. Inappropriate antibiotic prescriptions and lack of enforcement of prescription-only sales of antibiotics are both well-documented problems that Mexico shared with other Latin American countries [8,10]. Inappropriate antibiotic use and resistance have been very low on the government’s policy agenda, partly due to the lack of clear problem indicators, ignorance of strategies promoted internationally, and perception by policy makers that access to and supply of medicines should receive priority [6]. A White Paper by the Mexican Ministry of Health (MOH) proposing a National Pharmaceutical Policy in 2005 argued that regulating medicines sales to prescription-only by physicians – as stipulated by law since 1984 – had not been enforced as a measure to promote access to medicines because universal health care coverage has not been achieved [11].

In March 2010, the Mexican National Health Council announced a new policy enforcing the prescription-only status for antibiotic sales. It created an unprecedented public debate that gained prominence and space in the media. The announcement followed a declaration by the Minister of Health in May 2009 − in the midst of the H1N1 influenza outbreak − associating high mortality amongst influenza patients with self-medication, particularly with antibiotics [12]. The policy, enacted as a Ministerial Agreement, additionally required all antibiotic prescriptions to be retained and registered in pharmacies, and imposed high penalties for non-compliance, including rescinding business licenses. Soon after the announcement, the policy was opened to public discussion in a governmental website. Although several actors from the private sector declared opposition to the policy, it was finally approved with only minor changes, one of them delaying implementation for some months. The enforcement policy applied to 2,000 medicines – roughly one fifth of all medicines available commercially in the country. Drafting and implementing actions to enforce regulations on antibiotic sales were accompanied by press round-ups and press bulletins organized by diverse stakeholders, opinion columns in newspapers, discussion forums on the internet, and special programmes on radio and television. This paper analyzes media coverage of that policy process.

### Relevance of media coverage and interest group participation in the policy process

It has been recognized that the media not only have the potential to diffuse health information in society and to influence health behavior and attitudes [13,14], but also have the potential to influence public perceptions of health policy issues, the political elite’s policy considerations, and, eventually, the final policy product [15,16]. The media’s primary role in public policy making has been related to the process of agenda setting, through the ability to raise and shape issues and public opinion which, in turn, can impel governments to act [17]. The way in which issues are discussed in the media influences how problems are defined and, consequently, which policy alternatives are considered to address them. One manner in which the media can shape public opinion of health policy issues is by framing or emphasizing them in particular ways [18]. Although there is no consistency in how “framing” is referred to in the literature, a frame can be considered an emphasis on particular aspects of a topic [19]. Entman [20] defines framing as follows: “[to] frame is to select some aspects of a perceived reality and make them more salient in a communicating text in such a way as to promote a particular problem definition, causal interpretation, moral evaluation, and/or treatment recommendation.” Policy issues reported in the media depend on which facts are reported and which frames are chosen to address them. The choice of frame reflects journalistic conventions; the interaction among journalists and elite constituents, social movements, and scientific bodies; as well as a frame’s resonance with broader political values [19,21,22]. De Vreese [19] proposed distinguishing between “issue specific” frames and “generic” frames which are not related to a specific topic or a specific cultural or historical context. Actors use diverse frames to draw attention to issues of concern implying that certain types of solutions are called for [23]. Elite stakeholders have significant resources for shaping journalistic frames to serve their specific interests [21], which include reinforcing the status quo. However, the media can also give voice to less powerful stakeholders in order to reflect dissension or give a new direction to a policy debate. As Entman [20] explains, frames reveal the “imprint of power” when reflecting the identity of actors and interests competing to dominate news texts. Because framing emphasizes one aspect of a problem at the expense of others, it can restrict public debate on policies in the range of issues and policy alternatives considered [15,23].

The objective of this study was to analyze media coverage of the process that led to the enforcement policy on antibiotic sales regulations in Mexico focusing, firstly, on 1) Which themes gained more or less prominence; 2) Which stakeholder groups dominated the public debate; 3) What were their positions and actions vis-a-vis the enforcement policy. The results are discussed in relation to the processes of agenda setting, policy drafting, and policy implementation in order to highlight potential media and stakeholder effects on the overall development process. Secondly, with this study we aimed to shed light on the process of policy making for medicines in Mexico that could be relevant for other low and middle income countries (LMICs) wishing to develop policies directed at improving the use of antibiotics.

## Methods

### Data sources and collection

We conducted a systematic review of printed newspaper articles in Mexico using a media service that publishes daily on its website [24] all Mexican newspaper articles focussed on pharmaceutical issues. The service searches text electronically in 11 national newspapers in addition to seven in the capital district and other important cities. These newspapers represent the primary print media in terms of copies sold and political relevance in Mexico (see Table 1). The key words used for the electronic search include, among others, “medicines”, “antibiotics”, “Ministry of Health”, “pharmaceutical industry”, and “pharmacies”. We manually screened all of the newspaper articles published between January 2009 and December 2010 on the website and included in our study only those that covered issues related to antibiotic use or antibiotics sales regulation. The time period covers three stages of the policy process: 1) policy agenda setting (01 January 2009 to 24 March 2010); 2) policy drafting (25 March 2010 to 24 August 2010); and 3) policy implementation (25 August 2010 to 31 December 2010).

**Table 1:** Newspapers searched and number of articles included in the analysis

**Newspaper**	**Estimated number of copies sold per day**	**Number of articles included in the analysis**
*El Universal*	180, 000	45
*Reforma*	175, 000	37
*El Sol de México*	60,500	32
*Milenio diario*	40,000	30
*Excélsior*	90,000	24
*La Prensa*	150, 000	24
*Ovaciones*	82,082	21
*La Jornada*	38,759	19
*El financiero*	72,000	14
*El economista*	37,000	13
*Diario de México*	14,500	13
*El Gráfico*	300,000	11
*Uno más uno*	67,672	10
*La Crónica de Hoy*	70,000	9
*Impacto*	65,000	6
*Rumbo de México*	225,245	6
*La Razón*	61,075	4
*Metro*	143,000	4
Total number of articles		322

### Data analysis

In the identified newspaper articles, we conducted: 1) a quantitative content analysis to determine the frequency of topics and stakeholders covered; and 2) a qualitative content analysis to gain insight into stakeholder positions and actions in relation to the enforcement policy. We were interested in the content of the articles rather than in the presentation of the stories (such as headlines or graphical images). All articles were coded for the analysis. To develop the codebook, each author coded 20 articles identifying emerging themes through inductive reasoning [25]. Those themes were then structured and sorted into subject categories and each of the codes defined. The reliability and validity of the codification was checked by using investigator triangulation with 20 randomly selected newspaper articles. Discrepancies were discussed and adjustment made to the codebook (Table 2). All other newspaper articles were coded by one author using Atlas ti 5.2 software. In addition to the codebook, each article was coded according to stakeholders covered by the media: 1) government (executive); 2) congress (legislative); 3) pharmacies and outlet associations; 4) pharmaceutical industry; 5) medical associations; 6) civil society groups; 7) academic institutions; 8) journalists (in editorials and opinion columns); and 9) private sector other than the pharmaceutical industry. Finally, each time a stakeholder code was attached to a newspaper article, it was linked to the corresponding subject category so that we could link the stakeholder with the topics covered.

**Table 2:** Summary of the codebook

**Thematic category**	**Description of the category and subcategories included**
Self-medication (SM)	Purchase and use of prescription-only-medicines, including antibiotics, without medical prescription. Subcategories: i) SM to treat viral infections (influenza, other respiratory or gastro-intestinal infections); ii) causes and scope of SM; iii) solutions to the problem of SM, including the new policy to enforce sales regulation.
Antibiotic resistance (AR)	Antimicrobial or antibiotic resistance. Subcategories: i) causes and scope, including relation to self-medication and prescription ii) solutions, ii) using AR as an argument to support the new policy to enforce regulation on antibiotic sales.
Prescription (PM)	Medical PM of antibiotics. Subcategories: i) unjustified PM of antibiotics (for instance, for influenza and other respiratory diseases) ii) perception of low quality of PM; iii) need to train physicians and improve their PM of antibiotics
Economic impact (EI)	Direct and indirect EI resulting from the enforcement policy. Subcategories: i) EI in the private sector such as pharmacies and the pharmaceutical industry; ii) EI in the population.
Corruption (CO)	Corruption in the pharmaceutical sector derived from the enforcement policy. Subcategories: i) Black market of counterfeit medicines; ii) Black market of prescriptions; iii) CO in the process of pharmacy supervision; iv) physicians involved in CO.
Regulation (RE)	Regulation and policies related to the sales of antibiotics in Mexico. Subcategories: i) Legislative framework and lack of enforcement; ii) processes of drafting and implementing the enforcement policy; iii) need to develop an impact evaluation of the enforcement policy; iv) objective of the enforcement policy; v) need to disseminate information about the enforcement policy.
Health System (HS)	Functioning of the Mexican health system in relation to antibiotic use or the enforcement policy. Subcategories: i) problems of the HS; ii) impact of the enforcement policy on the health services provided in the HS; iii) access to health services for the population without health insurance.
Rational use of medicines (RUM)	Rational use of medicines in the national and international context. Subcategories: i) International guidelines on RUM; ii) recommendations to achieve RUM in Mexico; iii) causes and consequences of inappropriate use of antibiotics, including adverse reactions (but excluding references related solely to self-medication).
Pharmacies (PH)	Functioning of pharmacies in relation to antibiotic use or the enforcement policy. Subcategories: i) Operation of PH, ii) quality of services provided by pharmacy staff, and training of pharmacy staff; iii) position of pharmacy associations towards the enforcement policy; iv) demand of the pharmacy associations owners towards the government.

For the quantitative content analysis, we first calculated the frequency of thematic categories for each of the three time periods separately. If a thematic code was mentioned several times in the same article, we counted it only once. Secondly, stakeholder appearance was counted as the number of times a stakeholder was mentioned in a newspaper article: the same association or institution was counted only once per article even if it was mentioned several times in the same article. We counted separately different actors in the same stakeholder group mentioned in the same articles.

In order to understand the position and actions of stakeholders in relation to the new policy during each stage of the policy making process, a qualitative content analysis was performed of all the notes in which a stakeholder code appeared. Firstly, we concentrated the analysis on the voice of stakeholders, particularly quotes, rather than the voice of journalists (except for editorials and opinion columns for which we considered journalists as another stakeholder group). Here, we looked for statements about the policy and its objectives, and for arguments against or supporting the new policy. Secondly, we looked, both in the voice of stakeholders and journalists, for references to specific actions developed by the actors in relation to the new policy development. During the analysis, we searched for similarities and differences between actors within the same stakeholder group, and between different stakeholder groups.

## Results

**Figure 1:**
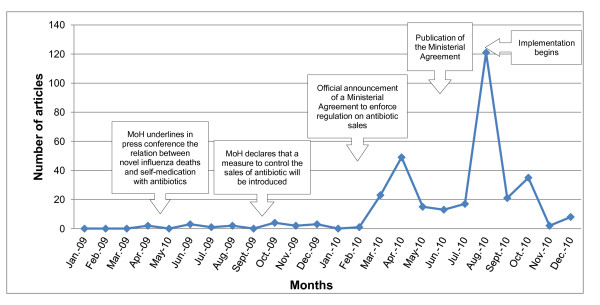
Monthly coverage on antibiotic use and regulation in Mexican printed media, and main policy milestones 2009-2010.

In total, 322 newspaper articles were included in the analysis; of them 18 were published during the agenda setting period, 185 during policy drafting, and 119 during implementation. Despite official statements regarding the problem of self-medication with antibiotics and the need to control antibiotic sales (following the influenza epidemic in 2009), these topics had little media coverage in the agenda setting period. During the study period (see Figure 1) we observed two peaks of media coverage on the topics of antibiotic use and antibiotic regulation. The first followed the public declaration by a member of the National Health Council, on 24 March 2010, announcing the Ministerial Agreement to enforce regulation of antibiotic sales. The second peak coincided with the start of the enforcement policy (i.e. the Agreement) implementation in August 2010. The newspaper sections in which the articles appeared were: front page or front section 36%; business or financial 11%; editorials and columns 9%; health or society sections 8%; politics 6%; others 30%.

### Topics covered by the news media

**Figure 2:**
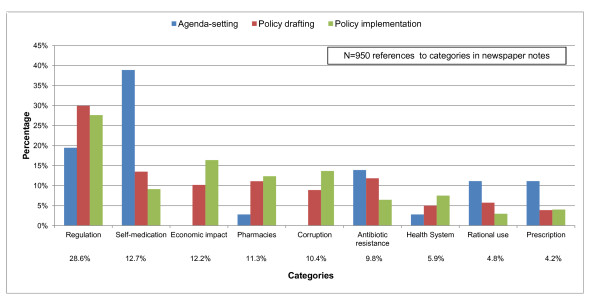
Theme categories covered by the printed media by stage of the policy process.

Overall, the most prominent themes found during the policy development process were regulation (29%), self-medication (13%), and economic impact (12%). Rational use of medicines, medical prescription, and health systems were least frequently mentioned. Figure 2 shows the prevalence of each theme category by stage during the development process.

*Agenda setting:* "Self-medication" was the most frequent thematic category, particularly the risks of inappropriate treatment of respiratory diseases, including H1N1 influenza, with antibiotics. The second most frequently mentioned thematic category was “regulation,” with most of the discussion focusing on the lack of enforcement for antibiotic sales. The third category, “antibiotic resistance,” was covered mainly as a consequence of self-medication. The need to promote the rational use of antibiotics – including improving medical prescribing – was also addressed during this stage.

*Policy drafting:* “Regulation” was the main thematic category during this phase, and included sub-categories such as the process of policy development (a third of the notes were coded in this category) and the objective of the enforcement. “Self-medication” and “antibiotic resistance” were frequently mentioned in relation to each other; both were used as arguments to support the new policy. The thematic category “pharmacy” was mentioned more frequently in relation to the role of pharmacies in implementing the policy, and to the demands made by the pharmacies to the government. The “economic impact” of the policy emerged as a theme: almost two-thirds of the notes mentioned the economic impact on the retail sector, while less than half referred to the economic impact on the population. “Corruption” was mentioned in relation to the risks of prescription falsification and counterfeit antibiotics subsequent to the policy.

*Policy implementation:* During this period, the theme “regulation” also dominated media coverage, but in relation to information and impact of the policy. Increasing media coverage included the categories “economic impact” on the retail sector, “corruption,” and “pharmacy”. The category “health system” also gained relevance, with most of the notes covering the impact of the enforcement on the health system and the inability of health system capacity to provide health care to the population. In contrast, discussions about the rational use of medicines became less prominent.

### Stakeholders covered by the media

**Figure 3:**
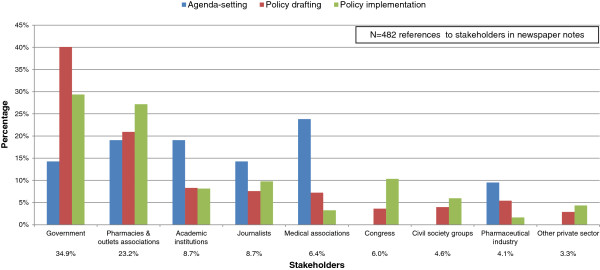
Printed media coverage of stakeholders by policy stage.

Figure 3 shows the frequency with which stakeholders were mentioned at each stage of the policy making process. Overall, the executive government dominated media coverage (35% of the notes), particularly the ministry of health (MOH) and the medicines regulatory authority (“COFEPRIS”), and to a much lesser extent other ministries. Second ranked were pharmacies and outlet associations (23%), particularly the association of independent pharmacies, “ANAFARMEX”. Editorials and opinion columns represented 9% of the notes. While academic institutions (9%), medical associations (6%), and the pharmaceutical industry (4%) had greater salience during the first two stages, their presence decreased in the last phase. In contrast, the coverage of legislators (6%), civil society groups (5%), and other private enterprises (3%, particularly consulting agencies) appeared later on in the policy process.

### Stakeholder positions and strategies

#### Ministry of health

The MOH, as the main supporter of the policy, focused initially on the dangers of self-medication, especially in relation to influenza, as the main justification for the policy. Among the first declarations published were: “*No more self-medication damaging the Mexicans*” [26]; “… *the use of antibiotics in cases of influenza H1N1 has been fatal* […]” [27]. Later, the problem of antimicrobial resistance was also mentioned, linking it only to self-medication; as the Health Minister explained: “*The regulation is not a caprice. In Mexico, the population is generating a resistance to antibiotics which arises from self-medication*” [28].

When faced with arguments by other actors against the policy and the demand to delay its implementation, the MOH defended the enforcement arguing that access to medical care was guaranteed with “Seguro Popular,” a recently-introduced form of health insurance. Even the Mexican President was quoted supporting the policy [29]. Later, the MOH recognized the arguments regarding counterfeit prescriptions and the lack of an information campaign, declaring that they would be addressed. Arguments that could have been used to gain support for the policy, such as protecting against adverse drug reactions and unnecessary expenses, were scarcely addressed.

When, because of policy implementation, physician consultation offices attached to pharmacies began to emerge, the position of the MOH was portrayed as vague, and only some statements cautioned the population about the quality of these consultations. In contrast, the MOH stressed the “*positive*” impact of the policy, declaring that a 35% reduction in antibiotic sales had been achieved [30].

#### Pharmacies and outlet associations

The position of these groups – as portrayed by the media – was predominantly against the enforcement policy. Even though initial statements were generally favourable (recognizing the problems of self-medication and antibiotic resistance), later these groups appeared as leading opponents. Main arguments conveyed were economic losses and logistical difficulties for the pharmacies (now forced to pursue additional tasks when selling antibiotics) as well as the negative health and economic effects particularly on poor populations with scarce access to healthcare. Other arguments were that the regulation would foster corruption, namely a black market in antibiotics and counterfeit prescriptions, as well as false inspectors blackmailing pharmacies.

Early strategies used against the policy included creating alliances (among some independent pharmacy associations, medicine distributors and outlet associations) demanding the government delay implementation in order to develop first a detailed impact assessment. Later they demanded a public education campaign (to change the “*culture of self-medication”*) and the provision of information systems for pharmacies to facilitate the new dispensing procedures. The association of independent pharmacies ANAFARMEX asked the government to stop the “*witch hunt”* against them and demanded the provision of loans to modernize small pharmacies, as well as the publication of a comprehensive database of all registered physicians in Mexico to detect false prescriptions. Another association (UNEFARM) organized a protest at the regulatory agency building against the regulation and sent a petition to the President. In contrast, declarations by large pharmacy chains were very scarce.

During the implementation period, ANAFARMEX demanded that the government permit sales of analgesics and cold and cough preparations only in pharmacies (and not in other outlets) as a way of compensating for economic loss. A later strategy developed by large pharmacy chains buffered the impact of the regulation by offering a “discount” on antibiotics, and creating physician offices attached to pharmacies to offer cheap or free medical consultation. Independent pharmacy associations pointed out that these “*express clinics*” generated unfair competition for smaller pharmacies. Six months after the implementation, four pharmacy chains plus some independent pharmacies had included consultation offices within drugstores; one large pharmacy chain declared that each of its 920 branches had physicians who offered free “*medical counseling*” for over 30,000 consumers each day [31].

#### Medical professional associations and academic institutions

According to the media, these stakeholders were predominantly in favor of the policy, highlighting antimicrobial resistance and adverse drug reactions as serious public health problems. At the same time, they stressed the need for an integrated action plan to improve medical prescribing, increase public awareness regarding the prudent use of antibiotics, and professionalize pharmacies. Some medical associations mentioned concerns about limited access to antibiotic prescriptions in poor rural communities and the quality of care offered in clinics attached to pharmacies.

During the policy drafting period, the action taken by these groups included the publication of open letters to the MOH and dissemination of a policy brief suggesting priority actions. Excerpts of the policy brief were later incorporated into the declarations of the MOH and in the official publication of the Ministerial Agreement.

#### Congress

Congress ─the Mexican legislative body─ declarations were scarcely mentioned in the media during the initial phase and reflected divided positions by political parties. While legislators of the ruling political party were supportive of the policy, members of opposing parties stressed the adverse health and economic impact on the population (now “*forced to pay a doctor*”) given the insufficient availability and quality of public health services. Some legislators in the Congress tried to promote an agreement to revoke the policy, arguing that it was “*a restrictive measure that did not correspond with a health policy for a country facing a political, economic and social crisis*” [32]. Strategies of the Congress to intervene included the proposal of a law reform to introduce higher punishments for those who use false prescriptions, and demands to the executive branch of the government to develop information campaigns and an independent evaluation of the policy impact.

#### Pharmaceutical industry and other private firms

During the early policy stages, media coverage addressed the declarations by some national pharmaceutical companies regarding administrative uncertainty around the policy change, as well as demanding the government develop an impact assessment and delay implementation. The generic medicines industry underlined the importance of prescribing antibiotics by generic name.

The statements by transnational pharmaceutical companies and consulting agencies were more frequent during the implementation period and focused on the high value of the antibiotic market in Mexico, the resultant *“reconfiguration of the market,”* the economic losses that the pharmaceutical sector would face and the need to *reinvent* the pharmacies. Deloitte, a private consulting agency, suggested that among the *new business models* to avoid economic drawbacks for pharmacies were the “in-situ physicians” [33]. They also warned that other regulations to control sales of other medicines could be expected. Except for one company, these groups omitted the issue of antimicrobial resistance.

#### Patients and civil society groups

There were only a few declarations by patients and civil society organizations. Although the media reflected the views of citizens (in letters to the editors, interviews to the population, and on-line discussion forums), the declarations of only one association (“El poder del consumidor”) were reflected in the media. This association underlined the possible negative effect of the enforcement policy for the poorest populations and stressed the need to improve access to health care, provide the public with more information and monitor user fees for private medical services.

#### Journalists

During the formulation period, several editorials and columns discussed the appropriateness of the regulation in recognizing the conflict between the problems of self-medication and antimicrobial resistance on one side, and the problem of low access to and low quality of public medical services (including long waiting times and medicines stock outs) on the other. Some columns questioned the real reasons for the regulation. Interestingly, many editorials were also critical about the lack of transparency and clear leadership in the process of policy making. Some editorials also questioned the feasibility of implementing the regulation in a country inundated by medicines promotion, where traditionally the law is not enforced, and there are too few inspectors to supervise the pharmaceutical sector.

## Discussion

The results highlight a number of important points about media coverage and stakeholder participation in relation to antibiotic use and the process of regulating antibiotic sales in Mexico that could be relevant for other LMICs aiming to develop policies directed to improving the use of medicines.

Even though the theme of regulation dominated media coverage, the focus was primarily on the *problems of developing* the policy, and little, except for self-medication, about its *objectives or its relation* to public health issues. The other dominant themes in media debates mirrored the voice of the two major stakeholders involved. On one hand, the MOH defended the policy by citing the dangers of self-medication with antibiotics; on the other, pharmacy associations opposed it, citing issues of economic impact and corruption. These divergent frames were scarcely addressed by the media on the common ground of a wider public debate on rational use of medicines and pharmaceutical policy. The emphasis on the problem of self-medication probably minimized public concerns about new physician offices attached to pharmacies and contributed to the belief that, with the policy, problems in antibiotic use had been solved. This represented a missed opportunity to discuss in-depth pharmaceutical policies and the development of a national strategy on antibiotic use. Additionally, media coverage also represented a missed opportunity to sensitize the public about the problem of antibiotic resistance and the need to use antibiotics prudently, which coincides with other studies which stress the scarcity of key mobilising information on health directed to the public [34].

Besides the interests and resources of stakeholders, the predominant themes reflected in news coverage of antibiotic use and antibiotic regulation can also be explained by journalistic conventions. These conventions tend to favour certain generic frames such as “episodic framing” [19] in which social issues and public affairs are portrayed as limited to events only, and not discussed in a broader context (e.g. coverage centred on the policy enactment, rather than in the problem of antibiotic use). Other common generic frames found in our study are “conflict” (winners and losers, corruption) and “economic consequences”. Coverage might also be explained by a regional trend characteristic of Latin America of health news reporting leaning towards frames of political conflict [35].

The present study also sheds light on the actors and processes involved in health and pharmaceutical policy making in Mexico. The executive government, mainly the MOH and the medicines regulatory authority dominated media coverage. This agrees with studies portraying the process of public policy making in Mexico as state-centered, with low levels of pluralistic debate [36]. The scant involvement of the legislature and the fact that coverage reflected contradictory views of individual legislators instead of that of an ad-hoc expert commission also coincides with other studies highlighting the traditionally weak role of the legislature and its advisory commissions in decision-making [36,37].

The associations of small independent pharmacies mostly affected by the regulation were successful in organizing press round-ups to push their views in media debates and make specific demands to the government. These groups also took advantage of this momentum to advance their own agenda, such as the threat of competition by pharmacy chains. This coincides with other authors pointing out that, although Mexico has attained a media establishment relatively independent of government control, it has also been increasingly beholden to commercial interests [38,39]. However, even when these association groups had a strong voice, their effect appears to be limited, suggesting that the influence of political factors may outweigh media framing effects on shaping policies, as other studies have pointed up [15].

Large pharmacy chains had a minor presence in media debates, as did the transnational pharmaceutical companies because their interests might not be greatly affected by the policy. Some large chains buffered the impact of the policy by opening new physician offices, which might have favored the prescription of branded medicines. Private enterprises largely framed the policy debate as a business issue, rather than a health issue, which resulted in a focus on maximizing profits rather than improving medicines use. This highlights the inherent complexities of pharmaceutical policies competing with both economic and public health interests.

Academic institutions and medical associations also took advantage of the policy momentum to push their own agendas, especially during the agenda setting and the early drafting periods. They used the media to stress the need to develop a comprehensive strategy on antibiotic use, a theme that had little resonance in the media, and had little impact on the final policy.

The scarcity of newspaper coverage describing dialogue or interaction between interest groups and the MOH, and the lack of governmental response to the demands of interest groups (such as developing a public information campaign), reflect a political context characterized by vertical and closed decision-making. In addition, remarks in the media and editorials speculating about the true reasons for the enforcement suggest a lack of transparency and lack of trust in the authorities.

Traditionally, civil society has had a very marginal role in decision-making in Mexico [36,37] with the voice of these actors barely present in media coverage. The absence of opinions by pharmacist associations can be explained by the small number of pharmacists working in private sector pharmacies. The large majority of pharmacy staff lack university degrees and undergo very little professional training in pharmaceutical sciences or related areas [40]. Hence, pharmacy associations with a business focus rather than professional associations emphasizing professional credentials dominated the media debate.

Our results coincide largely with the findings of an analysis of printed media coverage of tobacco policies in Mexico [41] which reported the overwhelming presence of governmental actors, and limited inclusion of academic centers and civil society organizations. However, because the tobacco study included coverage of the development of two laws, the presence of actors from the legislative branch was significantly larger. Interestingly, our results on actor participation contrast sharply with a similar study on medicines policy developed in Canada [15] where civil society was the dominant voice, demonstrating the strategic use of the media by these groups. Although Mexico is undergoing a democratic transition, the experience of media advocacy by academic and civil society groups is still developing.

Some lessons can be drawn from our study which are relevant for other low and middle-income countries aiming to develop policies directed at improving antibiotic use. First, active opposition by some stakeholders to the enforcement of regulations on antibiotic sales should be expected, particularly arguing the economic impact on the population. Actions seeking to counteract the policy goals include opening physician offices next to pharmacies. Second, even if the voices of governmental and business-related groups are favored in media coverage, the policy views of other groups can also achieve visibility in the media which could contribute to a more informed policy development process. Third, this study showed that even if governmental action focuses on a single problem and a single intervention (enforcement of sales regulations to avoid self-medication), other related issues will soon arise in public debate. These issues include access to medicines (a very sensitive issue for the population), the quality of medical services, the tracking of prescriptions and verification of their authenticity, and the adverse impact on commercial interests. These concerns highlight the need to discuss and develop interventions within the common framework of a pharmaceutical policy, and to engage stakeholders during the policy process. Finally, the scarcity of in-depth reporting on issues of antibiotic use and regulation in Mexico and Latin America has also been related to the limited availability of specialized journalism and independent information sources [39]. To address these limitations and to generate political priorities on antimicrobial use and resistance, the South American Infectious Disease Initiative [42] has worked with the news media in three countries, resulting in an improvement on the quantity and quality of coverage of these issues [39], an experience that might be worth replicating in other countries.

How does the Mexican case compare with other relevant experiences? The cases of India and Brazil, both middle-income countries that undertook antibiotic policies contemporaneously to Mexico are worth discussing. During 2010, international attention turned to the spread of the New Delhi metallo-beta-lactamase 1 (NDM-1) gene, believed to have originated in India, which causes several types of bacteria to become resistant. The naming of the gene and a description of its spread from that country to Europe in an influential international medical journal was followed by an intense public debate in India [43]. Controversy triggered action and, in 2011, the Indian government published the National Policy for Containment of Antimicrobial Resistance. The policy included, among many other interventions, the banning of over-the-counter antibiotic sales. However, later the government decided to put this regulation on hold indefinitely, arguing concerns over access to medicines, particularly in rural areas [44]. In the case of Brazil it was the spread of the multi-resistant bacterium CRKP (Carbapenem-Resistant *Klebsiella pneumoniae*), also followed by the media, which gave place to the resolution RDC 44/2010 of the Brazilian regulatory agency, regulating antibiotic sales to medical prescription only, to be retained at pharmacies. The resolution, supported by medical groups, faced the opposition of pharmacy associations, citing scarce access to medical services for the poorest populations, as well as the risk of triggering a parallel antibiotic market [45]. Taken together, the cases of these three countries underline the relevance of focusing events (influenza outbreak, NDM-1 and CRKP spread) to place issues of antibiotic use and resistance on the governmental agenda. However, these cases also show that an apparently technical issue (regulating antibiotic sales) can be very politically sensitive – because economic interests are affected and because of the relation to wider complex societal issues – underlying again the need to discuss antibiotic policies within the common framework of a pharmaceutical policy, and to mobilize political support.

Our study presents some limitations. First, our analysis used a unique coding scheme (“issue-specific” instead of “generic” frames) which limits our ability to generalize and compare the results [19]. However, whenever possible, we discussed our coding scheme in relation to generic frames. Other limitations concern excluding an analysis of electronic media (radio, television and internet) which are important in Mexico and other countries in the region [39] as well as the fact that our analysis was limited to the period of only five months after policy implementation. Relevant discussions and stakeholder actions that might have appeared later in the policy process or in other media outlets were excluded. Finally, as others pointed out, we can describe the representation of issues and stakeholder participation in the media, but we can only infer their *potential* effects on the public and on the policy process [15]. To provide a more comprehensive picture of stakeholder participation in the policy process it is necessary to use alternative data sources.

## Conclusions

Aspects of greater salience on media coverage were the issue of self medication used to justify the policy to enforce antibiotic sales regulations, and the negative aspects (economic impact and corruption) which largely represented the views of the two main stakeholders in the debate. Neither of these perspectives was conducive to public discussion on the wider problems of antibiotic misuse and resistance. Because framing emphasizes some aspects of the issues at the expense of others, the extensive coverage of the conflicts around developing and implementing the enforcement policy left little room to discuss wider societal issues, with the exception of discussions concerning access to medicines and health services. The absence of debate on the relationship between the policy and promoting rational use of medicines, improving the quality of services provided in pharmacies, and addressing the global public health threat posed by antimicrobial resistance meant a missed opportunity for discussing and developing a comprehensive national strategy on antibiotic use in Mexico.

## Competing interests

The authors declare that they have no competing interests.

## Authors’ contributions

AD and VJW conceived of the study and wrote the manuscript. EVV analyzed the data and drafted a first layout of the manuscript under supervision of AD and VJW. ST participated in the study design and data analysis. AD revised the entire manuscript which was approved by all other co-authors.

## Pre-publication history

The pre-publication history for this paper can be accessed here:

http://www.biomedcentral.com/1471-2458/12/1051/prepub
